# Pelvic Fractures in Children Results from the German Pelvic Trauma Registry: A Cohort Study

**DOI:** 10.1097/MD.0000000000002325

**Published:** 2015-12-28

**Authors:** Jörn Zwingmann, Emin Aghayev, Norbert P. Südkamp, Mirjam Neumann, Gerrit Bode, Fabian Stuby, Hagen Schmal

**Affiliations:** From the Department of Orthopaedic and Trauma Surgery, University of Freiburg Medical Center, Freiburg, Germany (JZ, NPS, MN, GB); Institute for Evaluative Research in Medicine, University of Bern, Bern, Switzerland (EA); Department of Traumatology and Reconstructive Surgery, BG Trauma Center Tubingen, Tubingen, Germany (FS); and Department of Orthopaedics and Traumatology, Odense University Hospital and Department of Clinical Research, University of Southern Denmark, Denmark (HS).

## Abstract

As pelvic fractures in children and adolescents are very rare, the surgical management is not well delineated nor are the postoperative complications. The aim of this study using the prospective data from German Pelvic Trauma Registry study was to evaluate the various treatment approaches compared to adults and delineated the differences in postoperative complications after pelvic injuries.

Using the prospective pelvic trauma registry established by the German Society of Traumatology and the German Section of the Arbeitsgemeinschaft für Osteosynthesefragen (AO), International in 1991, patients with pelvic fractures over a 12-year time frame submitted by any 1 of the 23 member level I trauma centers were reviewed.

We identified a total of 13,525 patients including pelvic fractures in 13,317 adults and 208 children aged ≤14 years and compared these 2 groups. The 2 groups’ Injury Severitiy Score (ISS) did not differ statistically. Lethality in the pediatric group was 6.3%, not statistically different from the adults’ 4.6%. In all, 18.3% of the pediatric pelvic fractures were treated surgically as compared to 22.7% in the adult group. No child suffered any thrombosis/embolism, acute respiratory distress syndrome (ARDS), multiorgan failure (MOF), or neurologic deficit, nor was any septic MOF detected. The differences between adults and children were statistically significant in that the children suffered less frequently from thrombosis/embolism (*P* = 0.041) and ARDS and MOF (*P* = 0.006).

This prospective multicenter study addressing patients with pelvic fractures reveals that the risk for a thrombosis/embolism, ARDS, and MOF is significant lower in pediatric patients than in adults. No statistical differences could be found in the ratios of operative therapy of the pelvic fractures in children compared to adults.

## INTRODUCTION

Trauma remains the leading cause of death in children.^1^ Injuries in the pelvic region in children and adolescents are rare: the incidence is between 2.4% and 7.5%.^2–4^ The main causes of injury are high-energy trauma^2^ associated with concomitant injuries to other regions (neurovascular and musculoskeletal structures, abdominal trauma, injuries to the central nerve system, etc.).^5^

A postmortem study of trauma patients showed a high rate of pelvic fracture-related deaths and a high incidence of pelvic fractures.^6–8^ An analysis from the American National Inpatient Pediatric Database revealed that children with pelvic injuries presented 5.2 concomitant injuries on average.^9^

A summary of the present literature shows that 83.3% of all pediatric pelvic injuries were due to high-energy trauma. The United States analysis also reveals that a pedestrian being struck by a car was the mechanism in 57.8%, a motor vehicle passenger was injured in 17.8%, a bicyclist in 4.9%, and a motorcyclist in 0.6%. A fall from a height was responsible for causing a pediatric pelvic fracture in 9.2%. Crush injuries (2.2%), injuries sustained during sport activities (2.1%), and farm accidents (0.5%) were uncommon.^10^ A key prognostic injury mechanism is the history of roll-over or crush (Injury Severitiy Score [ISS] up to 40 points, 86.6% associated injuries, 20% mortality rate >70% local complication rate).^11^

The ligaments of the children's pelvic are relatively stronger, and growth centers are present which together with the sacroiliac joints and pubic symphysis enable significant absorption capacity.^10^ Their pelvis is thus more elastic and more cartilaginous than that of adults.^12^ This elasticity results primarily in plastic deformation when the pelvic bone absorbs an impact^13^ which enables the pelvic anatomy's potential to be entirely restored, but not normally to the preinjury point. Due to this elasticity, the intrapelvic viscera are insufficiently protected, and intrapelvic organ injuries can occur in the absence of pelvic fractures or dislocations.^14^ Therefore, even simple or minimally displaced fractures are usually the result of a high-energy trauma, accompanied by the significant risk of additional intrapelvic and intraabdominal injuries.^14^ This leads to a relatively high incidence of isolated pubic rami fractures or iliac wing fractures.^15–25^

In contrast, complete disruption of the anterior and posterior pelvis or a complex pelvic injury can present a high risk factor for morbidity and mortality.^26,27^

Polytraumatized children should undergo computer tomography scans to rule out both pelvic fractures and associated nonmusculoskeletal injuries.^28^

The majority of pediatric pelvic fractures heal with no sequelae. Delayed union, pseudarthrosis, and persisting ligamentous instability are very rare.^29^ Complex pelvic traumas are associated in 31% of cases in conjunction with a higher rate of complications such as chronic back pain, persisting length discrepancy of the legs, difficulty urinating, and malfunction of the anal sphincter.^27^ Pelvic asymmetry can occur in children because of injury to the triradiate epiphysis; their mortality rate after pelvic fractures is around 5% (compared to 11%–18% in adults).^2,3^

The aim of this study based on a review of prospectively collected registry data was to evaluate the epidemiological data on children over a lengthy interval and to analyze how often and which emergency procedures were done to treat their pelvic fracture, as well as the postoperative complications these children suffered. Moreover, we were able to examine the clinical follow-up in a small subgroup of children to compare with a group of adults’ follow-up. Our a priori hypothesis was that there are relevant differences between the pelvic fractures in children compared to adults in terms of their epidemiological data, treatment methods, clinical outcomes, and types and rates of complications.

## METHODS

This study is based on data from the prospective pelvic trauma registry introduced by the German Society of Traumatology and the German Section of Arbeitsgemeinschaft für Osteosynthesefragen/Association of the Study of Internal Fixation International in 1991.^26,30–32^ The registry provides data on all patients with pelvic fractures treated from January 1991 to December 1993, from January 1998 to December 2000, and from January 2004 to December 2012 at any 1 of the about 23 level I trauma centers contributing to the registry. In the years missing between the 3 time periods, the register was inactive and no data were collected. Moreover, the number of contributing hospitals has changed overtime. Data acquisition and analysis were done in accordance with ethical guidelines and approved by our institutional review board. The trial was registered at the German Clinical Trials Register (DRKS no. 00000488).

Data were collected and processed using a standardized data sheet. For this purpose, we engaged a secured internet interface hosted by a professional academic provider (www.memdoc.org, Institute for Evaluative Research in Medicine, Bern, Switzerland). Registration occurred as soon as possible after the patient's admission and was updated consistently during follow-up by a trauma surgeon or study nurse. All selected items were exported from the original datasets into a Microsoft Excel (Microsoft Corp, Redmond, WA) document for the purpose of evaluation and statistical analysis. These items included age, gender, ISS, Hannover Polytrauma Score, fracture type, need for emergency measures, mortality, cause of death, and need for operative stabilization. The majority of the participating institutions (listed under Acknowledgments) fulfilled the requirements of a level I trauma center according to the classification of the American College of Surgery^33^ and German Trauma Society.^34^

All fractures were classified in each case by experienced orthopedic/trauma surgeons. Classifications were based on plain radiographs and computer tomography scans routinely used. Moreover, doubtful cases were discussed in regular meetings conducted by the working group on a 4-time annual basis to minimize interobserver bias. Pelvic ring fractures were classified using Tile classification system adopted by the Orthopaedic Trauma Association.^35^ Stable pelvic ring fractures were classified as type A, fractures with only rotational instability as type B, and fractures with both rotational and translational instability as type C injuries. We defined type B and C injuries presenting major visceral, neurovascular, or soft-tissue injuries as complex pelvic injuries.^36,37^ We assessed patients on whether they had sustained isolated pelvic ring fractures or pelvic ring fractures with additional injuries to other body regions. We applied the ISS and Hannover Polytrauma Score to assess the severity of injuries.^38,39^

The complications evaluated were divided into postoperative complications such as thrombosis and embolism, acute respiratory distress syndrome (ARDS), multi-organ failure (MOF), neurologic deficit (occurring during clinical treatment and not associated with the initial trauma; the point of time, or if an injury was surgery-associated not specifiable by the available data), postoperative bleeding and hematoma, wound infections, and seroma. The diagnosis of each complication was confirmed by a representative from that specific discipline (neurology, anesthesiology, and vascular specialist).

Whenever we had access to patients’ follow-up data, we applied the evaluated EuroQoL-5D (EQ-5D) and Merle d’Aubigné and Postel score.

The EQ-5D score measures disease-nonspecific quality of life based on the EQ-5D index (ranges from −0.6 to 1, where 1 is the best imaginable health); it is designed primarily for self-completion by respondents and is ideal for use in postal surveys. It is cognitively simple, takes only a few minutes to complete, and instructions to respondents are included in the questionnaire.^40^

The EQ-5D is suitable and validated for the use of pediatrics.^41^ Even though 2010 the EQ-5D-Y (a child-friendly version of the EQ-5D) was introduced the authors preferred to have on consistent questionnaire.^42^ There are no specific questionnaires for specific pelvic fractures in children, so the authors believe that this questionnaire was the best suitable for the investigation and was suitable and validated. Moreover, the clinical problems after pelvic fractures are very similar to patients suffering acetabular fractures.

The Merle d’Aubigné and Postel score is considered easy to understand and simple to administer and measures hip function via a score (ranges between 0 and 18, where 0 is the worst and 18 the best function).^43^

Our data were analyzed using WinStat 2009 (Bad Krozingen, Germany) for Microsoft Excel (Microsoft Corp, Redmond, WA) and statistical test as the Chi-square test and Mid-P-exact tests were used when appropriate. The alpha was set to 0.05 throughout the study.

## RESULTS

We identified a total of 13,525 patients including pelvic fractures in 13,317 adults and 208 children aged ≤14 years and compared these 2 groups. The time periods and numbers of adults versus children identified were: years 1991 to 1993 (n = 1722/57), years 1998 to 2000 (n = 2569/47), and years 2004 to 2012 (n = 9234/104).

**FIGURE 1:**
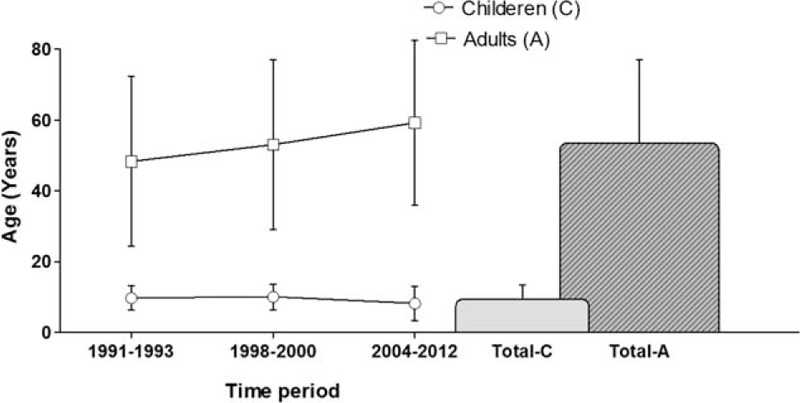
Development of mean age of the pediatric and adult groups since 1991. Mean age in the pediatric group was 9.3 years (±4.2) with a mean Injury Severitiy Score (ISS) of 16.7 (±15) points. Mean age in the adult group was 53.5 years (±23.5) with a mean ISS of 15 (±15) points (ISS: *P* > 0.05). The adults’ mean age rose over time (mean age: 48, 53, and 59 years) (*P* < 0.001), whereas we observed no statistical difference in the children's mean age overtime (*P* > 0.05).

Mean age in the pediatric group was 9.3 years (±4.2) with a mean ISS of 16.7 (±15) points. Mean age in the adult group was 53.5 years (±23.5) with a mean ISS of 15 (±15) points (ISS: *P* *>* 0.05). The adults’ mean age rose overtime (mean age: 48, 53 and 59 years) (*P* < 0.001), whereas we observed no statistical difference in the children's mean age overtime (*P* > 0.05) (Fig. 1).

**FIGURE 2:**
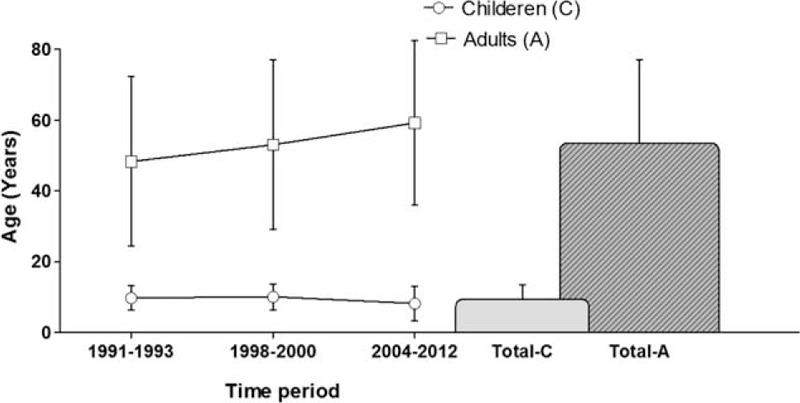
Development of the gender ratio of children and adults. We conducted a further analysis of the gender ratio of these patients, which revealed a continuous trend in the group of adults, namely a significantly decreasing gender ratio with a nearly equal ratio of 1.03 in the latest investigation period (*P* < 0.001). However, in the pediatric group, many more boys suffered pelvic fractures, at a ratio of 1.48 from 2004 to 2012 with an overall rate of 58% boys and 42% girls; there was no statistical difference in the gender ratio in the pediatric group's 3 time periods (*P* > 0.05).

We conducted a further analysis of the gender ratio of these patients and observed a continuous trend in the adult group, namely a significantly decreasing gender ratio with a nearly equal ratio of 1.03 in the latest investigation period (*P* < 0.001). However, in the pediatric group, many more boys suffered pelvic fractures – we noted a ratio of 1.48 from 2004 to 2012 with an overall rate of 58% boys and 42% girls; there was no statistical difference in the gender ratio in the pediatric group's 3 time periods (*P* > 0.05) (Fig. 2).

Table 1 summarizes the fracture classification in both groups, classified in Tile A, B, and C fractures,^35^ isolated acetabulum fractures (Iso Ac.), combined fractures of the acetabulum with an additional Tile A or B fracture type (Ac + Tile A/B), and complex fractures of the pelvis. Complex fractures were defined as pelvic fractures with additional injury to pelvic organs, vessels, and open fractures. The distribution of complex fractures among the children and adults was statistically not significantly different (*P* > 0.05). However, the distribution according to the Tile classification did differ significantly because of the much higher rate of acetabular fractures among the adults (*P* < 0.001) (Table 1).

**TABLE 1:**
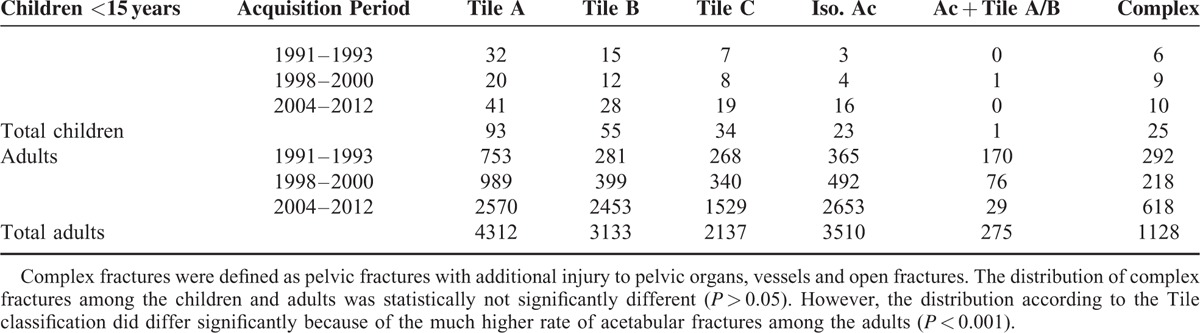
Summarizes the Fracture Classification in Both Groups, Classified in Tile A, B, and C fractures,^35^ Isolated Acetabulum Fractures (Iso Ac.), Combined Fractures of the Acetabulum With an Additional Tile A or B Fracture Type (Ac + Tile A/B) and Complex Fractures of the Pelvis

**FIGURE 3:**
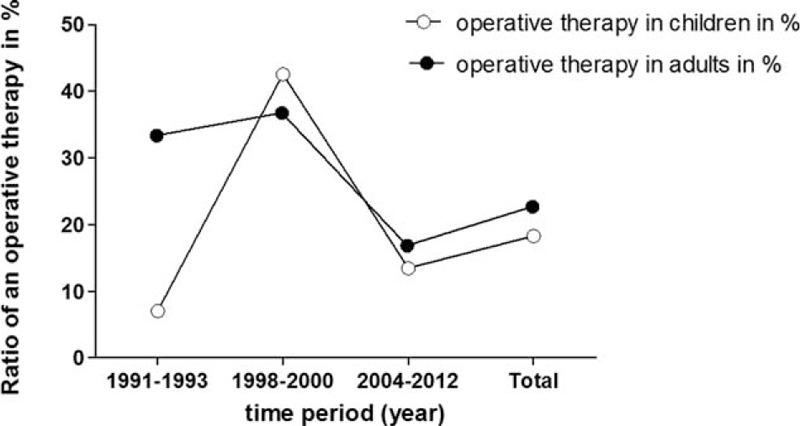
The ratio of an operative therapy of children and adults. In Figure 3 illustrates the ratios of operative therapy of the pelvic fractures. A total of 18.3% of the pediatric pelvic fractures were treated operatively, while 22.7% of the adults’ fractures were treated surgically. The other patients were treated conservatively; we detected no statistical difference between the adults and children in therapy terms (*P* > 0.05). What is remarkable is that in the very 1st time period, very few children underwent surgery compared to the adults. However, from the second time period, the rate of operative therapy is similar in both groups.

Figure 3 illustrates the ratios of operative therapy of the pelvic fractures. A total of 18.3% of the pediatric pelvic fractures were treated operatively, while 22.7% of the adults’ fractures were treated surgically. The other patients were treated conservatively; we detected no statistical difference between the adults and children in this regard (*P* *>* 0.05). What is remarkable is that in the initial time period (1/1991–12/1993), very few children underwent surgery compared to the adults. However, the rate of operative therapy from the 2nd and 3rd time period (1/1998–12/2000 and 1/2004–12/2012) is similar in both groups (Fig. 3).

Almost all the children who had undergone emergency surgery (15.4%) required subsequent therapy; the emergency interventions performed (in order of frequency) were:

1. Surgical emergency procedures with 8.7% external fixator (as “effective treatment” in 7.7%), 6.7% laparotomy, 1.4% emergency operation, and 0.5% pelvic C-clamp.
2. Not surgical procedures with 1% pelvic compression with a cloth, 1% pelvic binder.

A total of 18.8% of the adults in our survey had to undergo emergency surgery - their procedures were:

Surgical emergency procedures with 3% laparotomy, 1.9% emergency operation, 1.5% pelvic C-clamp, and 0.4% angioembolization.

We observed no statistical group difference in terms of emergency procedures (*P* < 0.05).

Table 2 summarizes the postoperative complications. No child suffered a thrombosis/embolism, ARDS, or a neurologic deficit. Two children suffered from “multi-organ failure” (MOF): one died after a severe craniocerebral injury and the other, after severe blood loss in the thorax and abdomen. These MOFs were thus not “septic” in nature. The differences between adults and children were statistically significant in terms of thrombosis/embolism (*P* = 0.041) and ARDS and MOF (*P* = 0.006). No children suffered a neurologic deficit, whereas 2% of the adults did (*P* = 0.015). No group differences were detected in terms of bleeding/hematoma or infect/seroma (*P* > 0.05) (Table 2).

**TABLE 2:**
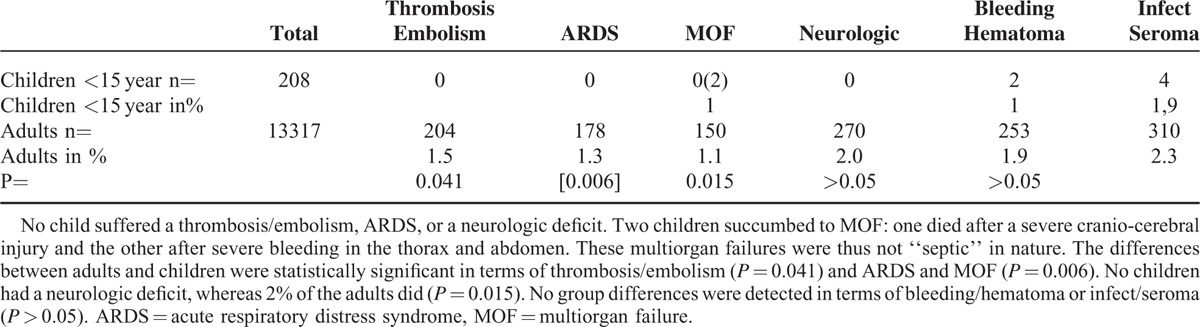
Summarizes Postoperative Complications

Lethality after suffering a pelvic fracture was not statistically significant, namely 13 children (6.3%) and 619 patients among the adults (4.6%) (*P* > 0.05).

**FIGURE 4:**
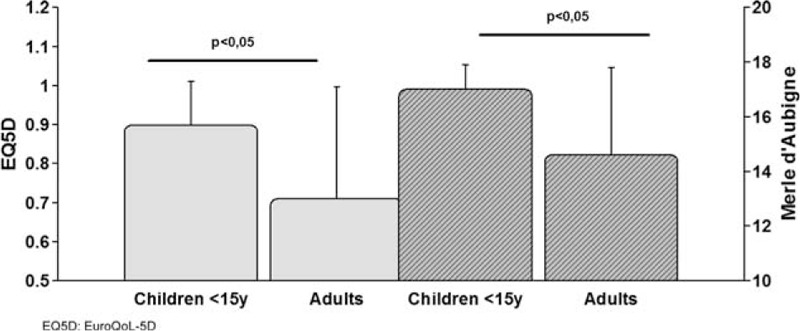
Clinical follow-up after pelvic fracture. Clinical follow-up is shown in Figure 4 and is based on data from 10 children (most from examinations in our clinic) 3.3 (±0.9) years after trauma and from available data in the registry of 631 adults who had undergone follow-up 2.5 (±1.7) years after trauma. The EQ5D score represents quality of life and the Merle d’Aubigne Score represents patient functionality. The 10 analyzed children had significantly better results in the follow-up investigations according to both scores (*P* < 0.05).

Clinical follow-up is shown in Figure 4 and is based on data from just 10 children (most from examinations in our clinic) 3.3 (±0.9) years after trauma and from data available from the registry of 631 adults who had undergone follow-up 2.5 (±1.7) years after trauma. The EQ5D score represents quality of life and the Merle d’Aubigne Score patient functionality. The 10 children we analyzed had significantly better results in the follow-up investigations according to both scores (*P* < 0.05) (Fig. 4).

## DISCUSSION

In investigating the prospective pelvic trauma registry introduced by the German Society of Traumatology and the German Section of Arbeitsgemeinschaft für Osteosynthesefragen/Association of the Study of Internal Fixation International, it was the aim of this study to analyze epidemiological results and differences between adults and children suffering a pelvic trauma in terms of emergency operative procedures, postoperative complications, and clinical follow-up examinations. According to a recently published review, only 1 publication^44^ has so far reported on more pediatric patients under investigation in a study.^45^ Gänsslen analyzed an overall mean age of 9 years in 10 analyzed studies and found a mean ISS of 15.7 points in 5 studies, and male predominance at a male/female ratio of approximately 1.4:1, all factors that resemble our investigation's findings.^10^ In analyzing the fracture types in different studies, Gänsslen cites a 60% to 80% rate of type A fractures, 10% to 35% type B injuries, and 10% to 16% type C injuries. Our results confirm his forecast for the German population in the last 2 decades. Type A are stable, type B are partially unstable injuries with partial posterior, rotational instability after antero-posterior or lateral compression, and type C are unstable injuries with combined anterior and posterior, vertical instability. Another commonly accepted classification of children's pelvic fracture is the Torode classification.^25^

For the children's analysis, we opted for the classic age cut-off at 14 years, as the epiphyseal plate in the acetabulum closes between 14 and 16 years.^46^ Mean age in the pediatric group we analyzed was 9.3 years (±4.2). Another review summarized 10 studies on pediatric pelvic fractures and identified a mean age of 9 years, similar to our data.^10^

Several alternatives and approaches for the operative treatment of pelvic fractures in children have been published, describing a wide (0.6%–30%) range of surgical interventions and reporting comparable rates of external and internal fixation.^10^ With the knowledge that conservatively treated displaced pelvic fractures in children can lead to pelvic asymmetry and poor clinical outcomes, more authors have focused on operative stabilization of the pelvic ring.^22,25,47–49^ The standard indications for the operative fixation of pelvic fractures are:

1. Concomitant therapy when open-wound treatment is necessary.
2. Additional hemorrhage control during resuscitation.^15^
3. Prevention of deformity in severely displaced fractures.^6,50–52^
4. The optimization and enhancement of patient mobility in particular situations (eg, polytrauma).

Therefore, only displaced fractures require surgical reduction and stabilization^15,25,43,52,53^ and only case descriptions are reported in the literature.^13^

Several emergency devices are currently available to stabilize an instable pelvis. Antishock trousers are no longer recommended in adults because of the high rate of complications.^54^ The application of pelvic slings, pelvic bed sheets, or a pelvic binder at the scene or in the emergency ward may be useful tools and treatment options for pediatric patients with an instable pelvic fracture.^55^ As our registry findings also reveal, stabilization with external fixation is the most common stabilization technique for pediatric pelvic fractures.^17,38,47,49,51,54,56,57^ After external fixation was applied, McIntyre et al^58^ detected a 60% rate of controlled bleeding in his cohort. The pelvic C-clamp is an adequate tool and can be used to stabilize the posterior pelvic ring, as another author mentions.^59^ Definitive reduction and internal fixation in acute management is only recommended when the patients are in stable condition. Feasible approaches for fixation are symphyseal plating, anterior plating of the SI-joint, and application of transiliosacral screws.^60^

Accepted methods to control pelvic hemorrhage are angiography or embolization and pelvic packing. Angiography and embolization to stabilize hemodynamics in pediatric patients with pelvis fractures can succeed, but reported time intervals between admission and the start of embolization range from 12 to 15 hours in an international study, and only 62 minutes in a German study.^61,62^ Another trauma-registry study reports the incidence of angiographic interventions as approximately 5% – a potential treatment strategy to stabilize hemodynamics.^4^ External fixation was the most often applied method in children and adults; however, the advantage at a younger age is that it is more frequently administered as definitive care.

Taking this study's data and the literature into account, external fixation seems to be an appropriate and minimally invasive treatment for most unstable pelvic fractures in children. Nevertheless, binding an unstable pelvic fracture (ie, in a preclinical or emergency room context) and angioembolization in the first hours of clinical stabilization are also treatment options for children.

We observed a significantly lower incidence of thrombosis, ARDS, and MOF in the pediatric group compared to adults. The “typical” complications in adults were almost nonexistent among the children.

The incidence of ARDS in the overall pediatric population is relatively low, with estimates ranging between 2.9^63^ and 12^64–66^ cases/100,000 children per year. The mortality rate in recently published studies from the USA and China are between 18% and 43% depending on the population and disease.^64,67^ Data from North America show mortality gradually dropping from 35% in the years 1996–1997 to 26% between 2004 and 2005.^67^ Even in very large studies, the main risk factors to develop an ARDS are pneumonia, aspiration, sepsis, near drowning, concomitant cardiac disease, and “others”; however, suffering a trauma or even polytrauma goes unmentioned.^68^

Venous thromboembolism (VTE) is an often-reported and major source of morbidity in critically ill trauma adult patients. In a pediatric population, Vavilala et al^69^ found that older children with high Injury Severity Scores, major vascular injury, craniotomy, or venous catheters are at risk for VTE.

Although trauma is noted as a risk factor in almost every reported series of pediatric patients with VTE, the rate of VTE specific to the pediatric trauma population is not well established. The reported incidence of VTE in the overall pediatric trauma population ranges from 0.02% to 0.33%^69–76^ and appears stable overtime.^71^

Evidence-based guidelines provide clear recommendations for VTE prophylaxis in adults who suffer major trauma.^77–79^ The initiation of low-dose unfractionated heparin or LMWH with intermittent pneumatic compression is recommended to begin within 24 to 48 hours of injury, unless contraindicated. In the presence of contraindications such as uncontrolled bleeding, presence of an epidural catheter, or severe coagulopathy, mechanical prophylaxis is suggested, and pharmacologic anticoagulation should begin once the bleeding risk has subsided. The recommended duration of VTE prophylaxis for patients with spinal cord injury is 3 months. Duration of prophylaxis is not clearly stated for other types of trauma; however, patients requiring major orthopedic surgery are recommended to receive prophylaxis for up to 35 days from the date of surgery, as opposed to only 10 to 14 days. For patients with isolated lower-leg injuries requiring leg immobilization, VTE prophylaxis is not recommended. Nor are screening ultrasounds recommended. The aforementioned guidelines make no recommendations for treating pediatric trauma patients.^18,77,79^

The data on medical or physical VTE prophylaxis in children and the newborn are insufficient.^80^ In adolescents in early puberty (≥Stadium Tanner II), the expositional and dispositional risk factors should be evaluated as they are in adults.^80–82^

Pelvic fractures in children are extremely rare, and they appear to becoming even less frequent over the most recent decades.

The fact that our study employs a prospective multicentre registry is both strength and weakness. On the one hand, including patients from several institutions best reflects a country's therapeutic reality. On the other hand, we relied on 21 to 27 active level I trauma centers contributing to the registry, and it goes without saying that treatment protocols depend on each institution's environment.

## CONCLUSION

The severity of injury seems to be similar in adults and children who suffer a pelvic fracture. In our registry patients, emergency procedures were performed in 15.4% of the children, and their risk for thrombosis/embolism, ARDS, and MOF was significant lower. Children seem to enjoy a better clinical outcome than adults according to the long time follow-up investigations we had access to. The reason for this probably has to do with poorly understood differences in the child's immune system.
