# Pelvic Fractures in Children Results from the German Pelvic Trauma Registry: A Cohort Study: Erratum

**DOI:** 10.1097/01.md.0000483155.73927.6c

**Published:** 2016-05-06

In the article “Pelvic Fractures in Children Results from the German Pelvic Trauma Registry: A Cohort Study”,^1^ which appeared in Volume 94, Issue 51 of *Medicine*, Dr. Schmal's institution was incorrectly listed in the original article. The article has since been corrected online.
